# Collecting Biospecimens From an Internet-Based Prospective Cohort Study of Inflammatory Bowel Disease (CCFA Partners): A Feasibility Study

**DOI:** 10.2196/resprot.5171

**Published:** 2016-01-05

**Authors:** Rachel L Randell, Ajay S Gulati, Suzanne F Cook, Christopher F Martin, Wenli Chen, Elizabeth L Jaeger, Alexi A Schoenborn, Patricia V Basta, Hendrik Dejong, Jingchun Luo, Marisa Gallant, Robert S Sandler, Millie D Long, Michael D Kappelman

**Affiliations:** ^1^Department of PediatricsDuke University School of MedicineDuke UniversityDurham, NCUnited States; ^2^Division of Gastroenterology and HepatologyDepartment of PediatricsUniversity of North Carolina at Chapel HillChapel Hill, NCUnited States; ^3^Center for Gastrointestinal Biology and DiseaseUniversity of North Carolina at Chapel HillChapel Hill, NCUnited States; ^4^Epidemiology Associates, LLCChapel Hill, NCUnited States; ^5^Division of Gastroenterology and HepatologyDepartment of MedicineUniversity of North Carolina at Chapel HillChapel Hill, NCUnited States; ^6^Department of EpidemiologyUniversity of North Carolina at Chapel HillChapel Hill, NCUnited States; ^7^Lineberger Comprehensive Cancer CenterUniversity of North Carolina at Chapel HillChapel Hill, NCUnited States; ^8^Department of PediatricsUniversity of North Carolina School of MedicineUniversity of North Carolina at Chapel HillChapel Hill, NCUnited States

**Keywords:** inflammatory bowel disease, biobank, Internet cohort, CCFA Partners

## Abstract

**Background:**

The Internet has successfully been used for patient-oriented survey research. Internet-based translational research may also be possible.

**Objective:**

Our aim was to study the feasibility of collecting biospecimens from CCFA Partners, an Internet-based inflammatory bowel disease (IBD) cohort.

**Methods:**

From August 20, 2013, to January 4, 2014, we randomly sampled 412 participants, plus 179 from a prior validation study, and invited them to contribute a biospecimen. Participants were randomized to type (blood, saliva), incentive (none, US $20, or US $50), and collection method for blood. The first 82 contributors were also invited to contribute stool. We used descriptive statistics and t tests for comparisons.

**Results:**

Of the 591 participants, 239 (40.4%) indicated interest and 171 (28.9%) contributed a biospecimen. Validation study participants were more likely to contribute than randomly selected participants (44% versus 23%, *P*<.001). The return rate for saliva was higher than blood collected by mobile phlebotomist and at doctors’ offices (38%, 31%, and 17% respectively, *P*<.001). For saliva, incentives were associated with higher return rates (43-44% versus 26%, *P*=.04); 61% contributed stool. Fourteen IBD-associated single nucleotide polymorphisms were genotyped, and risk allele frequencies were comparable to other large IBD populations. Bacterial DNA was successfully extracted from stool samples and was of sufficient quality to permit quantitative polymerase chain reaction for total bacteria.

**Conclusions:**

Participants are willing to contribute and it is feasible to collect biospecimens from an Internet-based IBD cohort. Home saliva kits yielded the highest return rate, though mobile phlebotomy was also effective. All samples were sufficient for genetic testing. These data support the feasibility of developing a centralized collection of biospecimens from this cohort to facilitate IBD translational studies.

## Introduction

Inflammatory bowel disease (IBD), including Crohn’s disease (CD) and ulcerative colitis (UC), affects 1.1-1.4 million individuals in the United States and is increasing in prevalence [1,2]. IBD imparts significant morbidity to patients [3] and burden to the health system [4,5]. The pathogenesis of IBD is related to a combination of genetic susceptibility, environmental factors, and host-microbial interactions in the gut [6,7]. Recent genome-wide association studies reveal at least 163 susceptibility loci for IBD [8], emphasizing the range and complexity of pathways that may be involved.

Despite this emerging knowledge, little is known about how these factors impact disease risk [9] and even less about disease course and exacerbations. Such knowledge is necessary to define prognosis and response to treatment, guide medical decision making and lifestyle modifications, and ultimately lead to personalized medicine for IBD. In fact, the recent Crohn’s and Colitis Foundation of America (CCFA) position paper on challenges in IBD identified studies to address these concepts as a top research priority [10].

Although case-control studies have historically been used for gene-environment studies, prospective cohort studies have many advantages, including the ability to study multiple outcomes [11] and critical evaluation of biological predictors of those outcomes. In fact, large prospective cohort studies with centralized biospecimen collection processes are considered “indispensable” by leaders in the field [12]. The Internet has the potential to be used to conduct gene-environment research remotely and at low cost with enhanced flexibility and rapidity, but to date it has not been widely utilized for these types of studies [13]. With the recent growing success of Internet-based cohorts and survey research [14-17], an opportunity to expand these cohorts to include biospecimen collection for gene-environment studies has now emerged.

CCFA Partners is an Internet-based cohort of over 13,000 adults with IBD that was developed in 2011 to accelerate clinical and patient-reported outcomes research [14]. Since its establishment, this cohort has been used in a number of cross-sectional and longitudinal studies covering a wide range of topics [10,14,18-22]. CCFA Partners has the potential to facilitate gene-environment and other translational studies, as well, if the cohort members would be willing to contribute biospecimens for molecular, genetic, and microbiological research. We previously surveyed over 1000 cohort members about their attitudes regarding biobanking, and an overwhelming majority (>90%) indicated willingness to contribute biospecimens [23]. However, little research exists on the practical aspects of collecting genetic or biospecimen samples from patients involved with Internet cohort studies.

Here, we report the feasibility of collecting saliva, blood, and stool from members of the CCFA Partners cohort in a systematic fashion for use in future studies. If feasible, this collection could provide a tremendous resource for IBD research and serve as a model for future methods of Internet-based translational research.

## Methods

### CCFA Partners

Methods for recruitment and prospective follow-up of participants in CCFA Partners have been previously described [14]. Inclusion criteria are ≥18 years of age, self-reported IBD, and Internet access. Participants complete a baseline survey upon registration and follow-up surveys every 6 months.

### Biospecimen Collection

Our study was designed to collect and analyze approximately 100 blood samples (50 by mobile phlebotomist and 50 drawn through physician offices) and 100 saliva samples. A total of 179 CCFA Partners participants who previously participated in a validation study [18] (“Validated population”), in which their physicians were contacted to confirm their IBD type and characteristics, were randomized to each of the three specimen categories (blood by mobile phlebotomist, blood at physician’s office, or saliva). Participants were also randomized to incentive level (none, US $20, or $50).

In addition to the validation cohort, we also randomized all CCFA Partners participants (“General CCFA Partners population”) taking any survey between August 20, 2013, and January 4, 2014, according to the same study arms. Within each arm, participants were successively invited until the recruitment target was approached.

Consent forms described the purpose, potential impact, and potential risks of genetic studies on biospecimens, as well as privacy protections including de-identification of samples, physical lock-and-key of stored specimens, and encryption of all data. Consenting participants were mailed a biospecimen collection kit either to be sent back to the Biospecimen Processing Facility or contacted by the mobile phlebotomy service to schedule a time and location for blood draw, as applicable.

Our study was designed to collect 50 stool samples among participants who provided genetic specimens. To achieve this, the first 82 participants who submitted a blood or saliva specimen were then invited to contribute a one-time stool sample. Participants were compensated US $20 for stool samples, regardless of whether they had been randomly assigned an incentive for the initial biospecimen.

For the mobile phlebotomy arm, we used Examination Management Services, Inc. (EMSI), a nationwide mobile specimen collection service. EMSI contacted participants to schedule a blood draw at a convenient time, and phlebotomists mailed blood samples directly to the Biospecimen Processing Facility per EMSI protocol. For the physician blood draw arm, we mailed each participant a kit containing blood draw supplies and a prepaid FedEx return label for overnight delivery. For the saliva collection arm, we mailed participants Oragene*-*500 oral collection kits (DNA Genotek, Inc.) with a prepaid FedEx Express saver return label. For stool, participants were instructed to ship stool samples on the day of collection with at least four -1 °C ice packs. All collection materials were affixed with a unique sample identification number and barcode, which was scanned when the specimen was processed by our lab.

### Host Genetic Analysis

DNA was extracted from saliva samples using the Chemagic Magnetic Separation Module I (MSMI) robotic system (Perkin Elmer), using the Chemagic DNA Saliva Kit and the MSMI 24-rod head. The MSMI system isolated DNA after cell lysis via highly specific binding of the DNA to proprietary M-PVA magnetic beads. Once bound, the DNA was washed several times and then released from the magnetic beads. Optical density readings were taken on a Nanodrop to assess the 260/280 and 260/230 ratio quality metrics. DNA quantitation was assessed via Picogreen using the Quant-iT PicoGreen dsDNA Assay Kit cat# P7589 (Life Technologies). DNA was extracted from blood using Puregene high salt extraction chemistry on the AutopureLS DNA extraction robotic system. DNA quantitation and 260/280 and 260/230 ratio quality metrics were performed on a Nanodrop spectrophotometer.

Saliva and blood samples were genotyped for 14 IBD-associated single nucleotide polymorphisms (SNPs) using TaqMan SNP Genotyping Assays from Life Technologies. We used pre-designed assays for all but one SNP (rs2066847), for which a custom primer was designed using previously established sequences (Forward primer: GTCCAATAACTGCATCACCTACCT; Reverse primer: CAGACTTCCAGGATGGTGTCATTC Probe 1 - VIC-MGB; Dye: CAGGCCCCTTGAAAG Probe 2 - FAM-MGB; Dye: CAGGCCCTTGAAAG) [24]. Polymerase chain reaction (PCR) volume was 5 uL.

### Fecal Microbial Analysis

Samples were aliquotted into cryovials and stored at -80 °C until the time of extraction. Bacterial DNA was extracted from 30-60 mg (solid) or 100-150 mg (liquid) of frozen fecal material as previously described [25]. Quantitative PCR was performed using primers for the 16S ribosomal ribonucleic acid (rRNA) gene of specific bacterial groups: forward, 5'-GTGSTGCAYGGYTGTCGTCA-3' and reverse, 5'-ACGTCRTCCMCACCTTCCTC-3', using 10 ng of DNA. Standard curves were generated using plasmids containing relevant PCR products for each bacterial group and used to enumerate copy number in individual samples.

### Data Analysis

We used descriptive statistics and *t* tests or Fisher’s exact test as applicable for comparisons between groups. All statistics were computed using SAS version 9.3. The study protocol was approved by the Institutional Review Board at the University of North Carolina at Chapel Hill.

## Results

### Study Population Characteristics

Of the 591 cohort member invited to contribute a biospecimen, 239 (40.4%) participants indicated interest and 171 (28.9%) contributed a biospecimen. In total, we collected 90 saliva samples, 47 blood samples from the mobile phlebotomy service, and 34 blood samples through physician offices. Demographic information for general CCFA Partners population included in this study and validated population participants is shown in Table 1. The general CCFA Partners population typically had lower education levels and a higher proportion of CD: 61.7% (254/412) versus 52.5% (94/179) CD for validated population. No significant differences were found across any other factors such as age, sex, race, or disease duration. Demographic factors for participants who indicated interest but did not contribute a specimen were compared to contributors (data not shown), and no significant differences were found.

**Table 1:** Study population characteristics stratified by random selection versus selection from prior validation study participants and by biospecimen contribution status.

		Selection status		General CCFA Partners population			Validated population		
		CCFA Partners general population (n=412)	Validated population (n=179)	Contributed (n=93)	Did not contribute^a^ (n=319)	*P*	Contributed (n=78)	Did not contribute^a^ (n=101)	*P*
Female, %		71.4	73.2	73	70.8	.67	74	72.2	.74
Age in years, mean		45.1	46.6	46.9	44.6	.49	48.2	45.4	.60
**Race, n (%)**						.54			.62
	White	365 (94.8)	156 (94.5)	81 (94)	285 (95.0)		68 (96)	88 (93.6)	
	Black/African American	8 (2.0)	4 (2.4)	1 (1)	7 (2.3)		1 (1)	3 (3.2)	
	Asian	1 (<1.0)	1 (<1.0)	0 (0)	1 (<1.0)		0 (0)	1 (1.0)	
	Other	11 (2.9)	4 (2.4)	4 (5)	7 (2.3)		2 (3)	2 (2.1)	
**Education, n (%)**						.82			.04
	12th grade or less	24 (6.0)	4 (2.4)	4 (4)	20 (6.5)		0 (0)	4 (4.2)	
	Some college	101 (25.6)	30 (18.0)	21 (24)	80 (26.1)		11 (15)	19 (19.8)	
	College	162 (41.0)	73 (43.7)	39 (44)	123 (40.1)		28 (39)	45 (46.9)	
	Graduate school	108 (28.0)	60 (35.9)	25 (28)	84 (27.4)		32 (45)	28 (29.2)	
**Disease type, n (%)**						.73			.14
	CD	254 (61.7)	94 (52.5)	59 (63)	196 (61.4)		46 (59)	48 (47.5)	
	UC/IC	157 (38.1)	84 (46.9)	34 (37)	123 (38.6)		32 (41)	52 (51.5)	
Disease duration in years, median		11.4	11.3	13	11.1		13	10.0	

^a^Includes participants who did not indicate interest and participants who indicated interest but never submitted a biospecimen.

### Demographic Factors Associated With Biospecimen Return Rates

Overall, age, sex, race, disease type, or duration were not related to contribution status. Participants from the validated population were twice as likely to submit a biospecimen than general CCFA Partners population: 43.6% versus 22.6% (78/179 versus 93/412, respectively), *P*<.001. Within this subgroup, higher education level was significantly associated with contribution status (*P*=.04) as shown in Table 1.

### Return Rates by Biospecimen Type and Incentives

**Figure 1:**
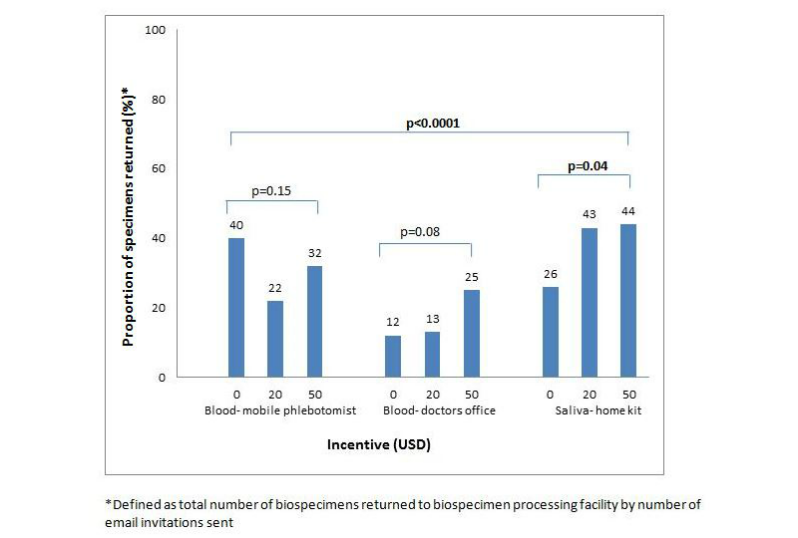
Proportions of biospecimens returned by collection method and level of incentive.

A total of 171 participants contributed blood or saliva. Four additional participants attempted to contribute, but for process reasons these were not obtained or biospecimen type was switched, so they were excluded from return rate analysis. Among biospecimen types, the return rate for saliva was higher than blood collected by mobile phlebotomist and at the doctor’s office (38%, 31%, and 17% respectively, *P*<.001) as shown in Figure 1. For saliva, US $20 and $50 incentive were associated with significantly higher return rate than no incentive: 43% (34/80) versus 26% (21/80), *P*=.03, and 43% (35/80) versus 26% (21/80), *P*=.05. For blood drawn at a doctor’s office visit, incentives typically showed a higher return rate, particularly the $50 incentive, but this did not reach statistical significance (*P*=.08). For blood collected by mobile phlebotomist, monetary incentive was not associated with an increased return rate. Of participants who submitted blood or saliva, 60% (49/82) also submitted a stool sample. There were no significant differences in stool contribution rates across general CCFA Partners versus validated population status (data not shown).

Return rates for each method and level of incentive were stratified by sex, prior participation in validation study, and race and education level as a proxy for socioeconomic status. An effect of incentives for saliva was observed in males, with 23% return rate for no incentive (5/22), 47% (9/19) for $20, and 58% (15/26) for $50 (*P*=.045). For females, the highest return rate for saliva of 43% was achieved with $20 incentive (26/61) but this was not statistically significant (*P*=.22). For saliva collection in participants who identified as white race, the $20 incentive yielded the highest return rate of 47% (34/76, *P*=.01,). There were no other significant differences in return rate across sex, prior validation study participation status, race, or education level (data not shown).

### Host Biospecimen Genotyping

A total of 171 samples were received (90 saliva, 81 blood). For saliva, total DNA yield ranged from 2.13-158.12 ug (median 52 ug) and 87% (81/93) of the samples yielded >20 ug. For blood, total DNA yield ranged from 6.59-382.14 ug (median 159 ug), 94% (76/81) of the samples yielded >50 ug, and 83% (67/81) yielded >100 ug. All samples were genotyped for 14 single nucleotide polymorphisms (SNPs) associated with IBD and risk allele frequencies (RAFs) were calculated. For all SNPs, the RAFs observed in our population were comparable to those in other large IBD populations [8,26] as shown in Table 2. Individual SNP frequencies for IBD overall, and for CD and UC, are provided in Multimedia Appendix 1. Crohn’s disease-associated SNPs like NOD2 (rs2066844, rs2066845, rs2066847) were more common in CD patients than UC patients. Of 2394 possible genotypes, 32 (1.3%) were undetermined. Of these undetermined genotypes, 53% (17/32) came from saliva and 47% (15/32) from blood samples.

**Table 2:** Risk allele frequencies for SNPs in the CCFA Partners cohort compared to other large IBD populations.

SNP	Notable genes	RAF	Reference^a^
rs12994997	ATG16L1	0.58	0.52
rs6426833		0.56	0.54
rs6017342	ADA,HNF4A	0.52	0.53
rs11209026	IL23R,IL12RB2	0.98	0.93
rs3024505	IL10,IL20,IL19,IL24; PIGR,MAPKAPK2; FAIM3,RASSF5	0.15	0.16
rs10761659		0.62	0.54
rs2155219		0.52	0.51
rs1893217		0.18	0.16
rs2413583	ATF4,TAB1, APOBEC3G	0.89	0.83
rs11564258	LRRK2,MUC19	0.03	0.03
rs2066844	NOD2	0.05	0.07^b^
rs2066845	NOD2	0.05	0.02^b^
rs2066847	NOD2	0.05	0.02

^a^RAF values obtained from [8].

^b^RAF values obtained from [26].

### Fecal Microbial Analysis

A total of 49 stool samples were received. Of these, 18% (9/49) were liquid stool. Total bacterial content ranged from 6.04x10^2^ to 4.97 × 10^6^ 16S sequences/mg stool, as shown in Table 3. Characteristics of individual stool samples are shown in Multimedia Appendix 2.

**Table 3:** Bacterial content of stool samples.

	Total bacteria, 16S sequences/mg stool (n=49)
Minimum	604
25% percentile	111,400
Median	436,000
75% percentile	683,500
Maximum	4,970,000
Mean	557,554
Standard deviation	754,369
Standard error of mean	107,767
Lower 95% CI of mean	340,874
Upper 95% CI of mean	774,234

## Discussion

### Principal Findings

These data show that participants from an Internet-based IBD cohort are willing to contribute, and it is feasible to collect, biospecimens in a centralized fashion for use in translational research. The highest return rates were obtained from home saliva kits, though a mobile phlebotomy service was also effective for collecting blood samples. Among study participants who contributed blood or saliva, stool collection is also feasible. All biospecimens collected provided sufficient quantity and quality of material for genetic or microbiological analysis. As over 6000 CCFA Partners participants complete 1 or more surveys each year, we estimate that, if taken to scale, the cohort could collect >1800 biospecimens with a 1-year period. Taken together, these findings suggest that the CCFA Partners cohort is a valuable resource for future translational research studies.

CCFA Partners participants who previously participated in a study to validate IBD diagnosis [18] were significantly more likely to contribute a biospecimen than participants from the general CCFA Partners population. This is likely due to the fact that by participating in the prior study, they had demonstrated that they were highly engaged research participants. Higher levels of education were associated with higher return rates within this subset, as well, indicating that there may be a particularly educated and motivated subset of the CCFA Partners cohort.

Our previous survey-based study of biobanking attitudes found that 39% of the surveyed cohort would “definitely” donate and 56% would “probably” donate biospecimens for research [23]. Our return rate of 29% out of all participants contacted for potential interest in this study is somewhat low in comparison. This discrepancy brings into question the validity and utility of hypothetical willingness surveys; however, differences in the response to a hypothetical and actual scenario are not entirely unexpected and practical or logistical concerns may have limited sample collection rather than lack of willingness. Findings from the willingness surveys could represent the highest proportion of participants that would contribute a biospecimen and thus could be used as a goal for overall rates of contribution. Additionally, our previous survey found that pharmaceutical funding negatively impacted stated willingness to contribute biospecimens [23]. As this pilot study was supported by industry, which was indicated on the consent form, this could also have negatively impacted our collection rates.

Our return rates for saliva were significantly higher than for blood or stool. A number of reasons could contribute to this finding. First, there may be a lower perceived burden of collecting saliva than blood or stool because it is self-collected, can be done at home, can be collected immediately, is not painful, and manipulation of saliva may seem cleaner, more hygienic, or more comfortable than the other options. Indeed, in our previous survey of perceptions of biospecimen collection, sample type preference favored saliva over blood or stool (94% versus 90% and 77%, respectively). As not all patients undergo routine bloodwork, this may explain the lower rates of DNA collection in the doctor’s office blood draw arm, as compared to the other arms.

The authors are unaware of any other publications on feasibility of collecting biospecimens from entirely Internet-based prospective cohort studies such as CCFA Partners; however, there is one cross-sectional Internet-based study of the feasibility of collecting both survey-based and biospecimen data in an elderly Welsh population [13]. The response rate for those with Internet access was approximately 40%, which is equal to the percentage of our population that indicated interest in the study. The return rate for biospecimens in the Welsh study was 75% for buccal swab and 70% for dry blood, which is equivalent to our biospecimen return rate of 72% for those who indicated interest in the study. Regarding collection rates by method of sample collection, our findings are also consistent with a prospective Nurse’s Health cohort study based in Denmark (not Internet-based) that reported a higher return rate for self-collected DNA samples (72-80%), either saliva or buccal cell samples, versus blood samples collected during an office visit (31%) [27]. Internet-based interventional studies have also met success with remote collection of biospecimens, reporting return rates of about 80% [28,29].

Our previous study on willingness to contribute biospecimens did not find that incentives were a reported motivator for participants [23]. In contrast, we found a significant effect of monetary incentive on saliva collection. We also found an effect of monetary incentive at the highest price point for blood collection with a doctor’s office kit, but this did not reach statistical significance. In contrast, the highest return rate for blood collected by mobile phlebotomy was with no incentive. Our finding that incentives were significantly associated with increased return rates of self-collected saliva specimens but not blood specimens collected by mobile phlebotomist or at a doctor’s office visit may represent a stronger effect of incentives on specimens that can be directly collected by participants. The discordant effects of monetary incentives on overall blood collection could suggest that participants who do contribute are intrinsically motivated, or that our degree of incentive was not high enough to overcome direct costs or perceived burden to the participants who did not contribute. Our findings are consistent with the results of a smoking cessation study with geographically dispersed participants in which the highest monetary incentive was associated with a higher return rate of self-collected buccal cell DNA biospecimens [30]. In a breast cancer genetics study, a small monetary incentive increased blood spot biospecimen return rates in breast cancer cases, but not controls [31], suggesting other factors that affect participation. Indeed, factors such as race [32,33], perceived trust [33], and chronic disease state [34] have been reported to affect participation in biospecimen research, although these findings are not replicated across different populations [35,36].

In all, monetary incentives at the highest price point may be a motivating factor for contributing biospecimens in the CCFA Partners cohort. Other patient-level factors such as demographics, chronic disease state, trust, and intrinsic motivation may play a more important role. For future studies, the cost-effectiveness of incentives should be weighed against perceived motivation within a specific population.

Across all modalities of biospecimen collection (home collection kits for saliva, mobile phlebotomy and doctor’s office kits for blood), we were able to obtain sufficient quantity and quality of genetic material for genetic analysis. Additionally, the SNP genotyping results show that the CCFA Partners population is representative of a large number of loci of interest in IBD research. These findings replicate previously established risk allele frequencies and known SNP associations, further supporting the utility of the CCFA Partners cohort for future genetic and translational studies. Stool samples in both solid and liquid form were sufficient for quantification of bacterial DNA and likely would be useful for microbiological and environmental studies of IBD.

### Strengths and Limitations

CCFA Partners has many strengths including the large size, prospective design, and entirely Internet-based platform, which allows for the largest known sample size for collecting patient-reported data in IBD. The prospective design also allows us to link patient-reported data, biospecimens, and biospecimen-derived data to future outcomes. Strengths specific to this biospecimen feasibility study include randomization across multiple strata including biospecimen type and incentive level and inclusion of all participants regardless of age or geographic location. Although we did target cohort members who previously participated in a study to validate IBD diagnosis, and therefore are more likely to be engaged and participate in this study, we analyzed return rates separately to eliminate selection bias. This group has now provided us with a repository of genetic and microbiological material in addition to detailed physician-validated information about their disease diagnosis, phenotype, and surgical history, which could be used for a variety of future translational research studies.

One limitation of this study is the relatively small sample size; however, this project was intended as a pilot and feasibility study. Nevertheless, there remains a possibility that larger numbers and greater statistical power would unmask other patterns in return rates, including differences by age, sex, race, disease type or disease duration, and the effect of incentives. While only four contributed biospecimens could not be obtained due to process factors (representing 1% of the sample size), this could represent a significant number or cost if biospecimens were to be collected on a much larger scale. By design, we attempted stool collection only among patients who provided genetic samples. While this allowed us to most efficiently estimate the proportion of participants who would provide both genetic and stool samples (an increasingly important aspect of translational IBD research), it did not allow estimation of the proportion of participants that would provide stool samples alone. Last, although CCFA Partners is a large IBD cohort and diagnoses have been validated [18], members tend to be highly educated and motivated, so these findings may not be generalizable to different IBD or other chronic disease populations.

### Conclusions

In conclusion, the successful collection and analysis of biospecimens from the CCFA Partners Internet-based cohort represents a tremendous opportunity for a wide scope of IBD research, including genetic, molecular, microbiological, epidemiological, clinical, and outcomes studies. Platforms such as CCFA Partners may provide important opportunities to translate basic science knowledge into clinically useful information, leading the way

toward precision medicine.

## Multimedia Appendix 1

Supplementary tables.

## Multimedia Appendix 2

Total bacterial content of stool samples in the CCFA Partners cohort.

## Abbreviations

CCFA: Crohn’s and Colitis Foundation of America
CD: Crohn’s disease
DNA: deoxyribonucleic acid
EMSI: Examination Management Services
IBD: inflammatory bowel diseases
PCR: polymerase chain reaction
RAF: risk allele frequency
SNP: single nucleotide polymorphism
UC: ulcerative colitis
