# Supplementary material for: Collecting Biospecimens From an Internet-Based Prospective Cohort Study of Inflammatory Bowel Disease (CCFA Partners): A Feasibility Study
Source: JMIR Res Protoc. 2016 Jan 5;5(1):e3. doi: 10.2196/resprot.5171 (PMC4719077; doi:10.2196/resprot.5171)
Supplement: Supplementary file 1 [file resprot_v5i1e3_app1.pdf]

Multimedia Appendix 1: Supplementary Tables

Appendix Table 1. Frequencies of Single Nucleotide Polymorphisms (SNPs) associated with IBD in the CCFA Partners cohort

SNP	Notable genes	Genotype	All IBD* (n)	CD (n, %)		UC (n, %)	
rs12994997	ATG16L1						
		Wild type (G, G)	28	9	8	17	28
		Heterozygous (G, A)	89	64	59	23	38
		Homozygous (A, A)	57	35	32	21	34
		Undetermined	0	0		0	
rs6426833							
		Wild type (G, G)	29	21	20	7	12
		Heterozygous (G, A)	94	56	52	35	58
		Homozygous (A, A)	49	30	28	18	30
		Undetermined	2	1		1	
rs6017342	ADA,HNF4A						
		Wild type (A, A)	41	29	27	11	18
		Heterozygous (A, C)	85	52	48	33	54
		Homozygous (C, C)	48	27	25	17	28
		Undetermined	0	0		0	

rs7517847	IL23R						
		Wild type (G, G)	27	10	41	16	26
		Heterozygous (G ,T)	71	43	65	21	34
		Homozygous (T, T)	76	47	67	24	39
		Undetermined	0	0		0	
rs11209026	IL23R,IL12RB2						
		Wild type (A, A)	0	0		0	
		Heterozygous (A, G)	6	2	2	3	5
		Homozygous (G, G)	166	104	98	58	95
		Undetermined	2	2		0	
rs3024505	IL10,IL20,IL19, IL24,PIGR, MAPKAPK2, FAIM3,RASSF5						
		Wild type (G, G)	124	74	70	45	75
		Heterozygous (G, A)	42	30	29	12	20
		Homozygous (A, A)	4	1	1	3	5
		Undetermined	4	3		1	
rs10761659							
		Wild type (A, A)	25	13	12	9	25
		Heterozygous (A, G)	80	56	52	22	37
		Homozygous (G, G)	67	38	36	29	48

		Undetermined	2	1		1	
rs2155219							
		Wild type (G, G)	35	22	22	12	21
		Heterozygous (G, T)	87	54	53	31	53
		Homozygous (T, T)	42	25	25	15	26
		Undetermined	10	7		3	
rs1893217							
		Wild type (A, A)	115	67	63	43	70
		Heterozygous (A, G)	52	36	34	16	26
		Homozygous (G, G)	5	3	3	2	3
		Undetermined	2	2		0	
rs2413583	ATF4,TAB1,						
	APOBEC3G	Wild type (T, T)	0	0		0	
		Heterozygous (T, C)	38	25	24	13	22
		Homozygous (C, C)	132	81	76	46	78
		Undetermined	4	2		2	
rs11564258	LRRK2,MUC19						
		Wild type (G, G)	161	100	94	57	93
		Heterozygous (G, A)	11	6	6	4	7
		Homozygous (A,	0	0		0	

		A)					
		Undetermined	2	2		0	
rs2066844, rs2066845, 2066847	NOD2						
		Wild type (any)	467	281	88	172	94
		Heterozygous (any)	46	34	11	11	6
		Homozygous (any)	5	5	2	0	
rs2066844	NOD2						
		Wild type (C, C)	153	89	85	59	97
		Heterozygous (C, T)	18	16	15	2	3
		Homozygous (T, T)	0	0		0	
		Undetermined	3	3		0	
rs2066845	NOD2						
		Wild type (G, G)	157	96	90	56	92
		Heterozygous (G, C)	13	8	7	5	8
		Homozygous (C, C)	3	3	3	0	
		Undetermined	1	1		0	
rs2066847	NOD2						
		Wild type (-,-)	157	96	89	57	93
		Heterozygous (C,-)	15	10	9	4	7

Homozygous (C, C)	2	2	2	0
Undetermined	0	0		0

*All IBD includes 5 additional individuals with indeterminate colitis phenotype

Appendix Table 2. Total bacterial content of stool samples in the CCFA Partners cohort

Sample #	Liquid stool (Yes/No)	Total Bacteria (16S sequences/mg stool)
1	No	1.25E+05
2	No	1.18E+06
3	No	9.88E+04
4	No	3.80E+05
5	No	1.12E+06
6	No	3.66E+03
7	No	6.59E+04
8	Yes	6.04E+02
9	No	9.15E+04
10	No	1.85E+06
11	No	7.18E+05
12	No	3.17E+05
13	No	6.55E+05
14	No	9.57E+04
15	No	9.91E+05
16	Yes	4.02E+05
17	No	5.11E+05
18	Yes	1.29E+04
19	No	8.05E+05

20	No	4.97E+06
21	No	4.56E+05
22	No	4.37E+05
23	No	7.12E+05
24	No	1.24E+05
25	No	3.35E+05
26	No	4.41E+05
27	No	4.36E+05
28	No	3.62E+05
29	No	5.53E+05
30	No	3.77E+05
31	No	8.33E+05
32	No	9.34E+05
33	Yes	5.79E+05
34	No	1.39E+06
35	Yes	2.14E+05
36	No	3.08E+05
37	No	4.25E+05
38	No	3.36E+05
39	Yes	5.25E+03
40	No	1.11E+04
41	No	8.17E+05
42	No	6.43E+05
43	Yes	5.48E+04
44	No	6.19E+05
45	No	5.63E+05

46	No	4.52E+05
47	Yes	4.84E+05
48	Yes	5.33E+03
49	No	2.06E+04
