# Supplementary material for: Collecting Biospecimens From an Internet-Based Prospective Cohort Study of Inflammatory Bowel Disease (CCFA Partners): A Feasibility Study
Source: JMIR Res Protoc. 2016 Jan 5;5(1):e3. doi: 10.2196/resprot.5171 (PMC4719077; doi:10.2196/resprot.5171)
Supplement: Supplementary file 2 [file resprot_v5i1e3_app2.pdf]

Appendix Table 2. Total bacterial content of stool samples in the CCFA Partners cohort

Sample #	Liquid stool (Yes/No)	Total Bacteria (16S sequences/mg stool)
1	No	1.25E+05
2	No	1.18E+06
3	No	9.88E+04
4	No	3.80E+05
5	No	1.12E+06
6	No	3.66E+03
7	No	6.59E+04
8	Yes	6.04E+02
9	No	9.15E+04
10	No	1.85E+06
11	No	7.18E+05
12	No	3.17E+05
13	No	6.55E+05
14	No	9.57E+04
15	No	9.91E+05
16	Yes	4.02E+05
17	No	5.11E+05
18	Yes	1.29E+04
19	No	8.05E+05
20	No	4.97E+06
21	No	4.56E+05

22	No	4.37E+05
23	No	7.12E+05
24	No	1.24E+05
25	No	3.35E+05
26	No	4.41E+05
27	No	4.36E+05
28	No	3.62E+05
29	No	5.53E+05
30	No	3.77E+05
31	No	8.33E+05
32	No	9.34E+05
33	Yes	5.79E+05
34	No	1.39E+06
35	Yes	2.14E+05
36	No	3.08E+05
37	No	4.25E+05
38	No	3.36E+05
39	Yes	5.25E+03
40	No	1.11E+04
41	No	8.17E+05
42	No	6.43E+05
43	Yes	5.48E+04
44	No	6.19E+05
45	No	5.63E+05
46	No	4.52E+05

47	Yes	4.84E+05
48	Yes	5.33E+03
49	No	2.06E+04
